# Potential of Seed Halopriming in the Mitigation of Salinity Stress during Germination and Seedling Establishment in Durum Wheat (*Triticum durum* Desf.)

**DOI:** 10.3390/plants13010066

**Published:** 2023-12-25

**Authors:** Manel Hmissi, Abdelmajid Krouma, Francisco García-Sánchez, Mohamed Chaieb

**Affiliations:** 1 Laboratory of Ecosystems and Biodiversity in Arid Land of Tunisia, Faculty of Sciences, University of Sfax, Sfax 3029, Tunisia; hmissiepkrouma@gmail.com (M.H.); mchaieb133@gmail.com (M.C.); 2 Faculty of Sciences and Techniques of Sidi Bouzid, University of Kairouan, Kairouan 3100, Tunisia; 3 Centro de Edafología y Biología Aplicada del Segura (CEBAS-CSIC), E-30100 Murcia, Spain; fgs@cebas.csic.es

**Keywords:** seed pretreatment, initial vigor, osmotic effect, seedling emergence, stress tolerance index, toxic effect

## Abstract

The salinity of soils and irrigation water is among the main factors that limit plant productivity worldwide. Several alternatives have been proposed to get around this problem. However, these alternatives have faced difficulties in their implementation. As an alternative, the adverse effects of salinity on crop yield can be minimized by selecting species and varieties better adapted to salinity and/or by finding priming agents that give plants a certain tolerance during the vegetative and reproductive stages. The latter are strictly dependent on germination and seedling establishment. For this purpose, a laboratory experiment was conducted on three Tunisian wheat cultivars (Karim, Razeg, and Maali) subjected to moderate salinity stress (MSS, 5 g L^−1^ NaCl), severe salinity stress (SSS, 10 g L^−1^ NaCl), or control (0 NaCl) after soaking the seeds in a solution of KNO_3_ or ZnSO_4_ (0.5 g L^−1^). Salinity stress significantly decreased germination capacity (GC) and induced osmotic stress under MSS, which declined under SSS in favor of toxic stress. Pretreatment of seeds with KNO_3_ or ZnSO_4_ alleviated the toxic effect, and seedlings recovered initial vigor and GC even under SSS. The Karim cultivar showed better tolerance to salinity and a higher ability to react to priming agents. The calculated sensitivity tolerance index (STI) based on germination capacity, seedling growth, and initial vigor decreased in all cultivars under salt stress; however, this parameter clearly discriminated the studied cultivars. Karim was the most tolerant as compared to Razeg and Maali. We conclude that halopriming provides a benefit by alleviating the harmful effects of salt toxicity and that cultivars differ in their response to priming and extent of salt stress. KNO_3_ and ZnSO_4_ effectively alleviated the inhibitory effect of salt stress on seed germination and seedling establishment while significantly improving initial vigor.

## 1. Introduction

Soil salinity is one of the main abiotic stresses limiting crop growth [1,2,3]. In addition to its natural origin, soil salinization is the result of poor irrigation practices and poor irrigation water quality [4]. Currently, out of 1.5 billion hectares of cultivated land worldwide, around 77 million hectares are affected by excessive salt content [5]. In Tunisia, soil salinization affects virtually all regions, particularly those with arid and semi-arid bioclimates (the central and southern parts of the country). Previous studies have reported that about 1.5 million hectares of productive agricultural systems are lost every year due to drought, the use of saline and non-conventional water, irrigation techniques, and cultivation practices [3]; at this rate, about 50% of arable land will be lost by 2050 [6]. Furthermore, even more losses can be expected in the face of the accelerated climate change observed in recent years.

Scientists and breeders have proposed several alternatives to preserve crop development, including the desalination of irrigation water and saline soils, the use of appropriate cultivation practices, and the selection of species and cultivars adapted to salinity. The first two solutions appear onerous and difficult to implement in light of their difficulty of application and high cost, while the third seems the most promising. Selecting these salt-tolerant species and cultivars is one way of mitigating the effects of saline soils, and offers the possibility of enhancing the value of brackish waters and marginal regions affected by salinity. In this context, searching for intraspecific variability is being pursued as well. Although the behavior of cultivated plants in saline environments has long been the subject of numerous studies, Tunisian durum wheat (*Triticum durum* Desf.) has not received particular attention. However, this rainfed and irrigated cereal, which is used for both human and animal consumption, is highly recommended due to its high resistance to salts [7]. The first requirement for a satisfactory crop is proper planting, and the success of the germination phase is decisive for the rest of the vegetative period. However, this phase has been shown to be sensitive to salinity, sometimes more so than the later phases of the plant cycle [7], and adopting an approach to improve its tolerance represents a successful alternative.

Seed priming is one of an efficient solutions that acts on the metabolic processes of germination by adapting them to stressful conditions. It is considered a fast, inexpensive, non-polluting, and easy-to-apply technique that improves plant tolerance to salinity from germination to yield. [7,8,9]. This technique allows for the regulation of cell functions from structural to genetic levels and improves antioxidant capacity during seed germination and seedling growth [10]. The improved role of certain phytohormones (auxins, gibberellins, abscisic acid, ethylene, jasmonic acids, etc.) as a response to seed priming was observed by Bruce et al. [11]. Seed priming allows plants to perform adequately under stressful conditions. Several natural, chemical, and synthetic compounds have been proposed as seed priming agents, such as silicon, ascorbic acid, salicylic acid, polyamines, KNO_3_, KCl, CuSO4, and ZnSO_4_, all of which act positively on the subsequent plant cycle by enhancing plant growth and development [7,12,13]. Li et al. [14] reported a positive effect of melatonin on germination and seedling characteristics under water stress. Sheteiwy et al. [15] demonstrated that jasmonic acid used for seed priming or shoot pulverization significantly improved the water relation parameters, chlorophyll biosynthesis, and photosynthetic activity in soybean seedlings grown under salinity stress. Sarkar et al. [16] demonstrated that bamboo seed priming with KNO_3_ significantly raised the germination capacity. The same priming agent added with urea promoted antioxidant defense and increased tolerance to salt and drought stress [17]. Otherwise, priming oat seeds with CuSO_4_ and ZnSO_4_ improved all growth traits compared to unprimed seeds. Based on their own studies, Iqbal et al. [18] concluded that seed priming could partly overcome the effects of salinity stress. Accordingly, Khan et al. [19] revealed wheat plant growth and antioxidant enzyme activity to be significantly improved after seed priming with ZnSO_4_. It has been demonstrated that the harmful effects of salinity on wheat can be overcome through seed priming with KCl [10].

In Tunisia, salty soils occupy about 25% of the country’s total arable land. Durum wheat is grown under irrigation (often with relatively saline water) on an annual area of 48,700 ha, producing about 180,000 tons [20]. The remaining 80% of national production is obtained by rain-fed cultivation, mostly in arid and semi-arid environments on relatively saline soils. Thus, it seems very important to study the interaction between seed priming and wheat cultivars as affected by salt stress along with its repercussions on germination and seedling traits. In this context, we set up the present study to evaluate the germination response of durum wheat cultivar seeds to salinity stress following treatment by various chemical agents. Aiming to propose efficient, rapid, and low-cost tools to improve durum wheat seed germination and tolerance in saline areas, we assessed the extent of factors controlling germination, their osmotic effect (OE) or toxic effect (TE), and their subsequent effects on seedling establishment.

## 2. Results

**Figure 1:**
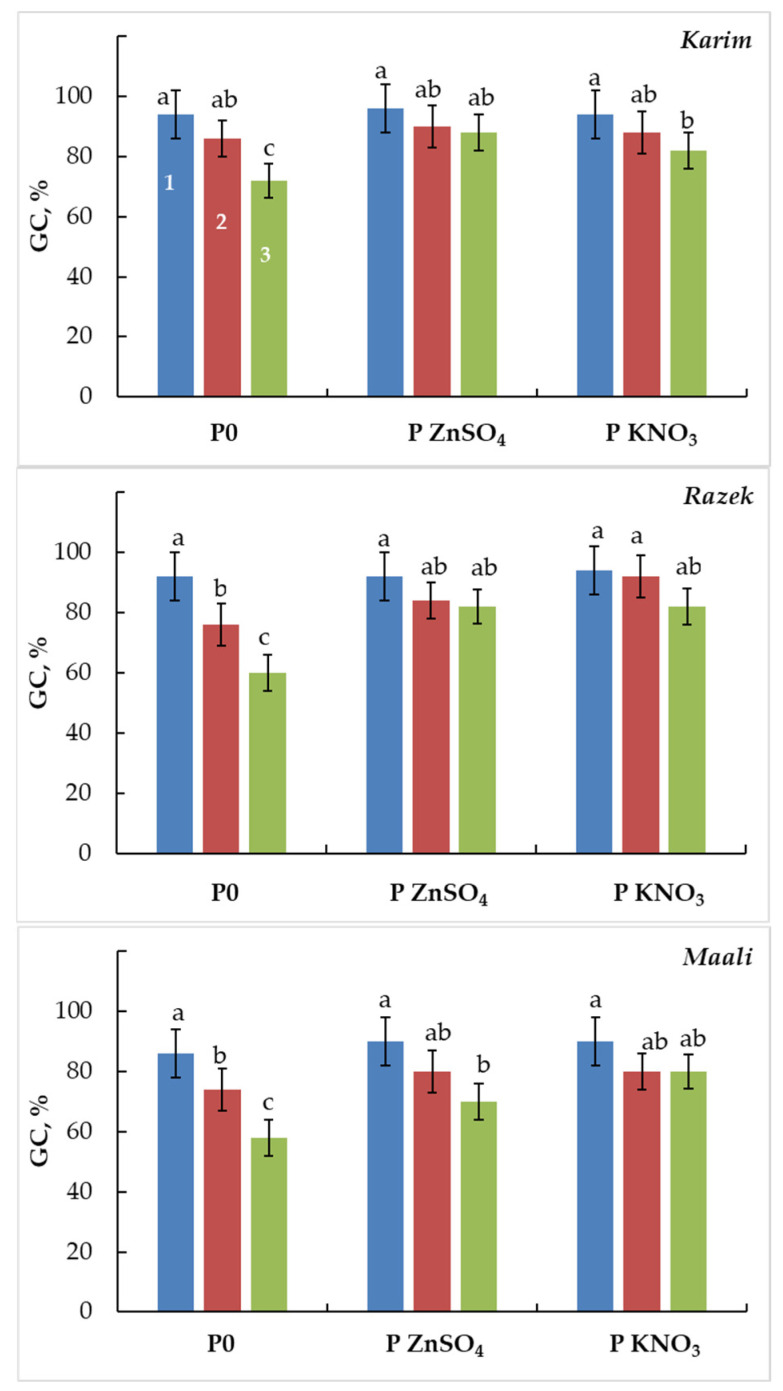
Germination capacity (GC) of durum wheat seeds primed with ZnSO_4_ (P ZnSO_4_), KNO_3_ (P KNO_3_), or not primed (P0) and subjected to different salinity stress levels (1: 0 g L^−1^ NaCl, 2: MSS = 5 g L^−1^ NaCl, and 3: SSS = 10 g L^−1^ NaCl). The significance of the differences is marked by the letters in the figures. Means with different letters are significantly different at α = 0.05 according to Fisher’s Least Significant Difference. The standard error of the mean of 100 seeds (five replicates of twenty seeds each) is represented by the bars on the columns.

With regard to stress severity and cultivars, salinity stress significantly hampers germination capacity (GC) in durum wheat (Figure 1). Under moderate as well as severe salinity, Karim remained the least affected as compared to the other cultivars. GC decreased by 9%, 17%, and 14% under MSS and by 23%, 35%, and 33% under SSS in Karim, Ragez, and Maali, respectively. However, seed pretreatment by ZnSO_4_ or KNO_3_ significantly attenuated the harmful effects of salinity (Figure 1). When pretreated with ZnSO_4_, the salt-inhibiting effect on GC decreased by 6%, 9%, and 11% under MSS and by 8%, 11%, and 22% under SSS in Karim, Razeg, and Maali, respectively. When pretreated with KNO_3_, the salt-inhibiting effect decreased GC by 6%, 2%, and 11% under MSS and by 13%, 13%, and 11% under SSS in Karim, Razeg, and Maali, respectively. Compared to unprimed seeds, ZnSO_4_ improved GC by 5% and 22% in Karim, 11% and 37% in Razeg, and 8% and 21% in Maali under MSS and SSS, respectively. For KNO_3_, these improvements fluctuated between 2% and 14% in Karim, 21% and 37% in Razeg, and 8% and 38% in Maali under MSS and SSS, respectively.

**Figure 2:**
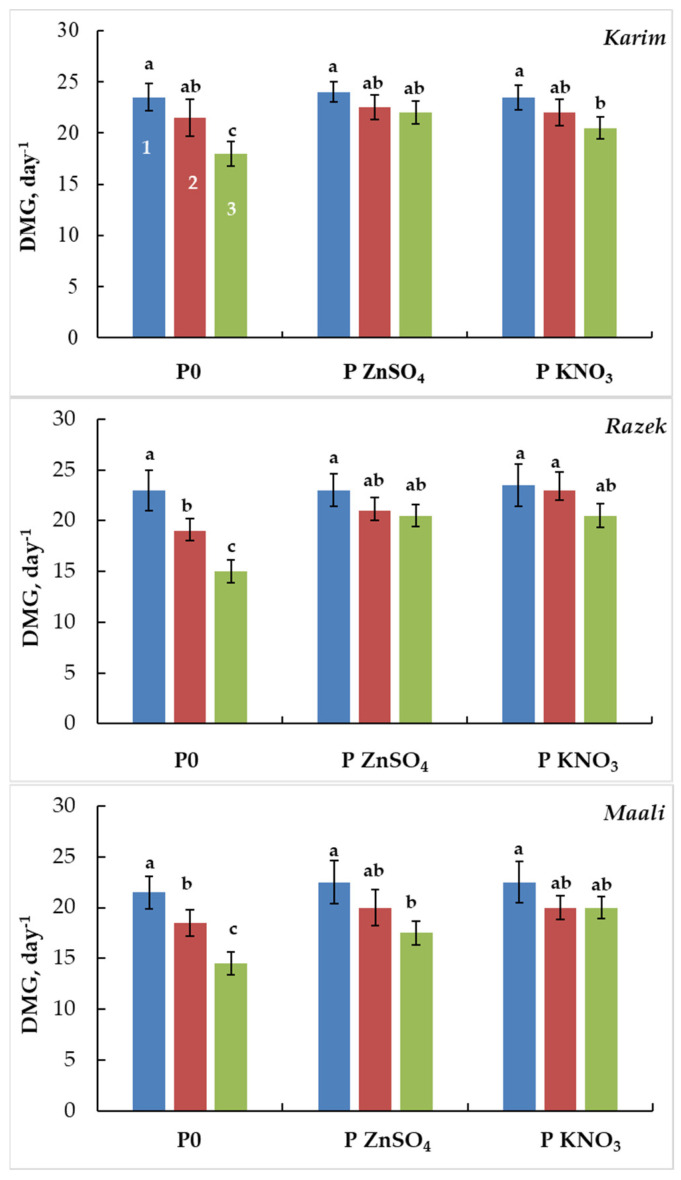
Daily mean germination (DMG) of durum wheat seeds primed with ZnSO_4_ (P ZnSO_4_), KNO_3_ (P KNO_3_), or not primed (P0) and subjected to different salinity stress levels (1: 0 g L^−1^ NaCl, 2: MSS = 5 g L^−1^ NaCl, and 3: SSS = 10 g L^−1^ NaCl). The significance of the differences is marked by the letters in the figures. Means with different letters are significantly different at α = 0.05 according to Fisher’s Least Significant Difference. The standard error of the mean of 100 seeds (five replicates of twenty seeds each) is represented by the bars on the columns.

The calculation of the daily mean germination (DMG) demonstrated that increasing salinity stress decreased DMG in all cultivars (Figure 2). However, Karim maintained a higher DMG under salinity stress compared to Razeg and Maali. The beneficial effect of seed priming was particularly apparent under SSS, where the decrease in DMG was mitigated (23% to 8% and 13% in Karim, 35% to 11% and 13% in Razeg, and 33% to 22% and 11% in Maali) after ZnSO_4_ and KNO_3_ pretreatment.

**Figure 3:**
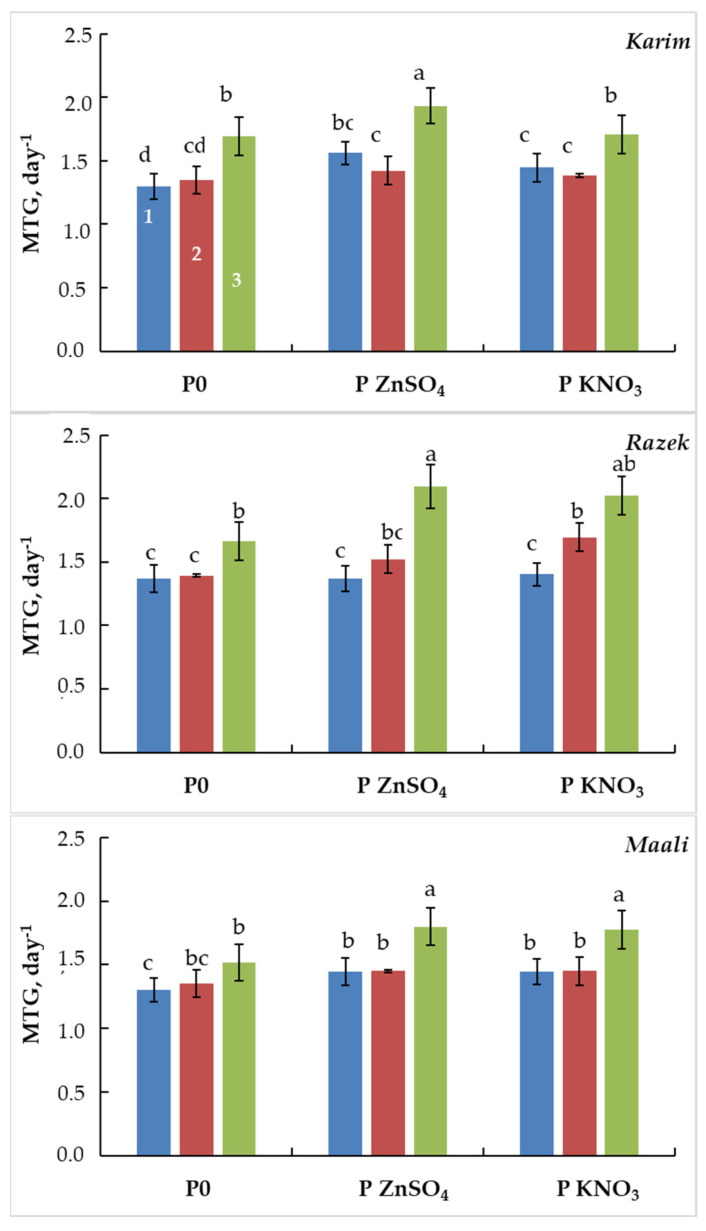
Mean time germination (MTG) of durum wheat seeds primed with ZnSO_4_ (P ZnSO_4_), KNO_3_ (P KNO_3_), or not primed (P0) and subjected to different salinity stress levels (1: 0 g L^−1^ NaCl, 2: MSS = 5 g L^−1^ NaCl, and 3: SSS = 10 g L^−1^ NaCl). The significance of the differences is marked by the letters in the figures. Means with different letters are significantly different at α = 0.05 according to Fisher’s Least Significant Difference. The standard error of the mean of 100 seeds (five replicates of twenty seeds each) is represented by the bars on the columns.

The mean time germination (MTG) is a parameter that reflects the germination speed. Under salinity stress, this parameter increased slightly in all genotypes under MSS (+2 to +4%) and significantly increased under SSS (+31% in Karim, +22% in Razeg, and +17% in Maali, Figure 3). When primed with ZnSO_4_ or KNO_3_, the decrease in MTG eased slightly in Karim (+24% and +18% instead of +31%), whereas it continues to increase in Razeg (+53% and +44% instead of +2%) and Maali (+25% and +23% instead of +17%).

**Figure 4:**
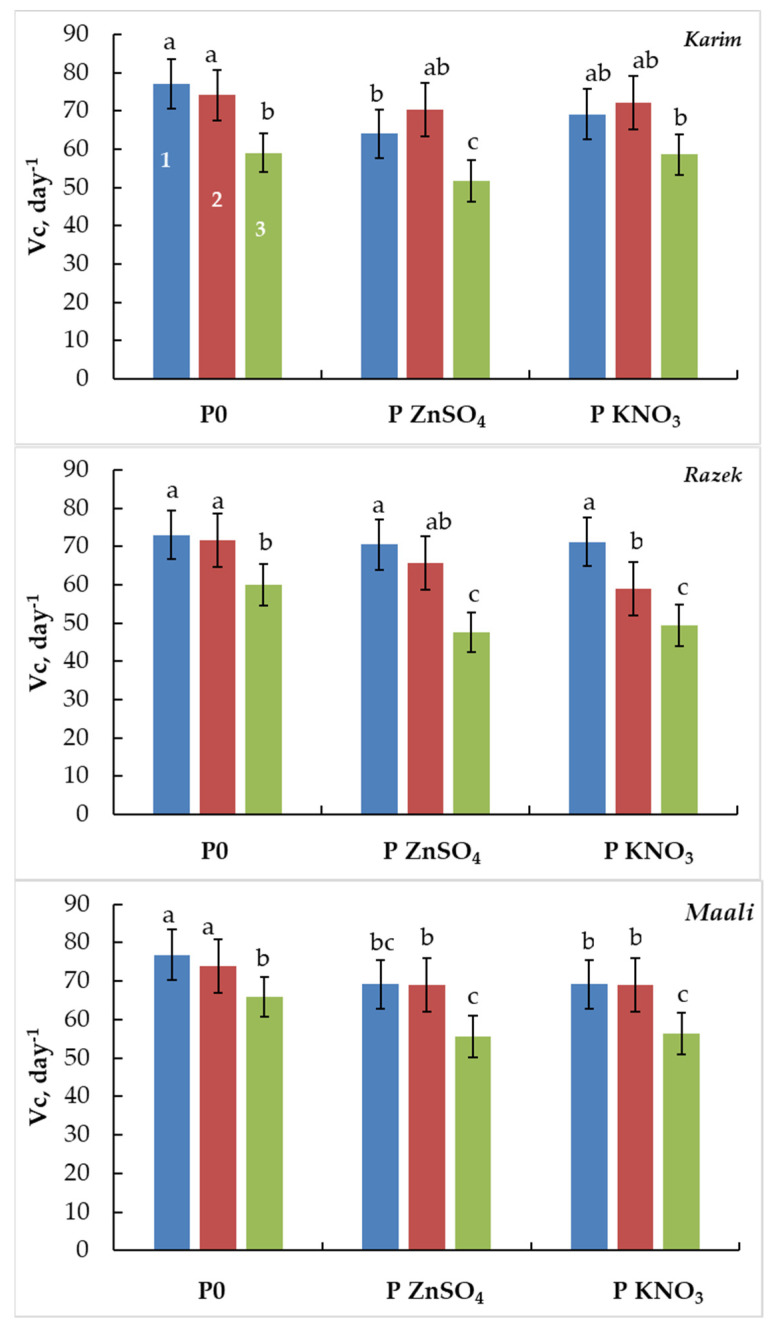
Velocity coefficient of durum wheat seeds primed with ZnSO_4_ (P ZnSO_4_), KNO_3_ (P KNO_3_), or not primed (P0) and subjected to different salinity stress levels (1: 0 g L^−1^ NaCl, 2: MSS = 5 g L^−1^ NaCl, and 3: SSS = 10 g L^−1^ NaCl). The significance of the differences is marked by the letters in the figures. Means with different letters are significantly different at α = 0.05 according to Fisher’s Least Significant Difference. The standard error of the mean of 100 seeds (five replicates of twenty seeds each) is represented by the bars on the columns.

The velocity coefficient (Vc) is another trait of the germinating process. Vc decreased in line with salinity stress severity in all cultivars (Figure 4). This decrease is cultivar-dependent but significant only under SSS (−23%, −18%, and −14% in Karim, Razeg, and Maali, respectively). Independently of the applied agent, seed germination did not modify the Vc under MSS and attenuated the inhibitory effect of salinity on the germinated seed number and germination time under SSS.

**Figure 5:**
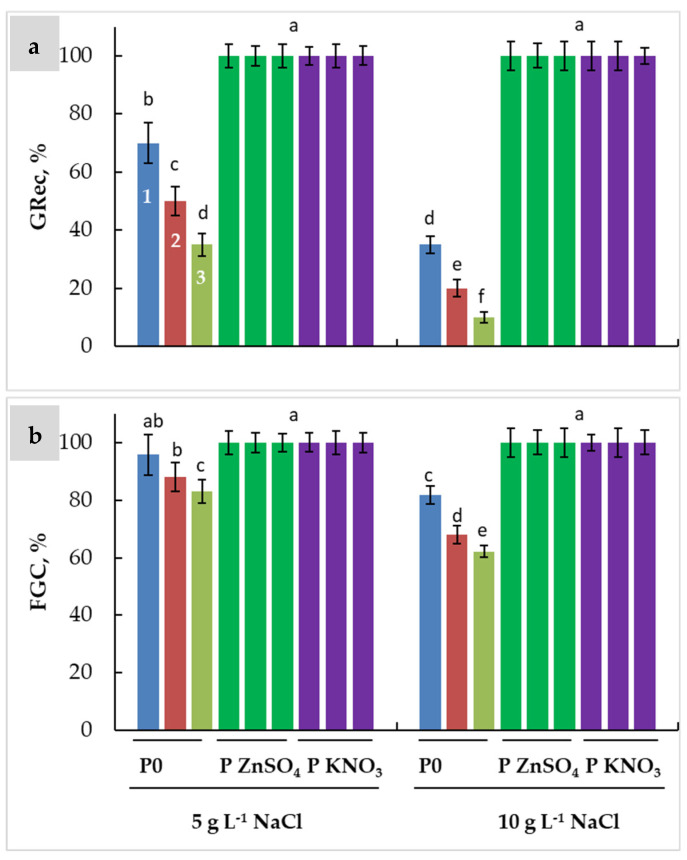
Germination recovery (GRec, (**a**)) and final germination capacity (FGC, (**b**)) of durum wheat seeds primed with ZnSO_4_ (P ZnSO_4_), KNO_3_ (P KNO_3_), or not primed (P0) and subjected to different levels of salinity stress (5 g L^−1^ NaCl, and 10 g L^−1^ NaCl). The significance of the differences is marked by the letters in the figures. Means with different letters are significantly different at α = 0.05 according to Fisher’s Least Significant Difference. The standard error of the mean of 100 seeds (five replicates of twenty seeds each) is represented by the bars on the columns. 1: Karim, 2: Razeg, 3: Maali.

Regarding all these parameters highlighting the deleterious effects of SSS on wheat seed germination and the beneficial effects of ZnSO_4_ and KNO_3_ as priming agents, we calculated the germination recovery (GRec) after transfer of non-germinating seeds under salinity to deionized water. This operation allows the limits of the osmotic and toxic effects of NaCl to be identified. The obtained results demonstrated that unprimed seeds express the ability to germinate when transferred to deionized water in all cultivars (Figure 5a). Karim expressed higher GRec (70% under MSS and 35% under SSS) compared to the other cultivars (50% under MSS and 20% under SSS in Razeg and 35% under MSS and 10% under SSS in Maali). When primed, GRec reached its maximum (100%) in all cultivars independently of the priming agent (Figure 5a). Thus, when GRec was added to the GC, the final germination capacity (FGC) reached a value that clearly exceeds the initial GC (96% and 82% instead of 86% and 72% in Karim, 88% and 68% instead of 76% and 60% in Razeg, and 83% and 62% instead of 74% and 58% in Maali; Figure 5b), while the maximum FGC reached 100% in all cultivars.

**Figure 6:**
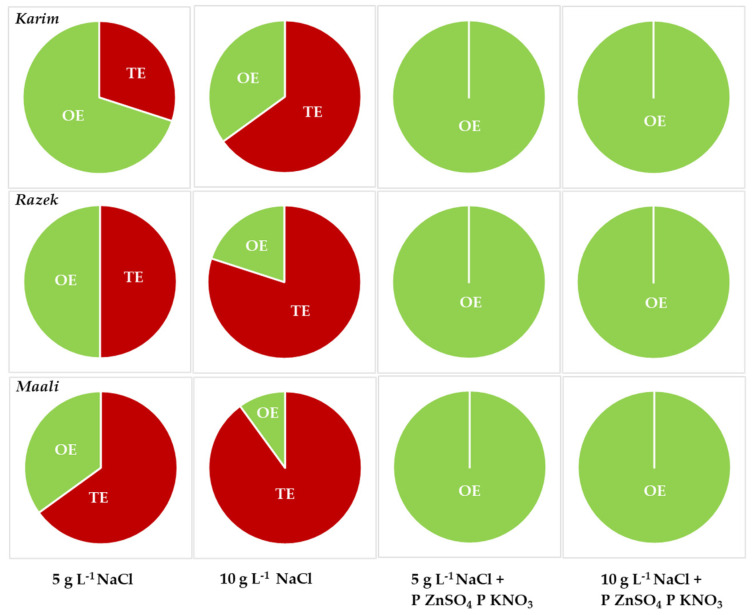
Osmotic (OE) and toxic (TE) effects of salinity stress affecting durum wheat seed germination (5 and 10 g L^−1^ NaCl) and when primed with ZnSO_4_ or KNO_3_ (P ZnSO_4_, P KNO_3_).

The inhibitory effect of salinity on seed germination is known to be linked to the toxicity of the Na^+^ ion, its various effects on cell membrane and metabolism, and/or resulting osmotic problems with repercussions on water uptake. When representing the extent of the OE and TE, we noticed that the deleterious effects of moderate salinity stress on seed germination are governed by the OE in Karim and to a lesser extent by TE (OE ≥ TE). In contrast, for Razeg the OE and TE under MSS were approximately equal, and for Maali TE > OE. The toxic effect predominated under SSS in all cultivars (Figure 6). However, seed priming alleviated TE in favor of OE (100%), eliminating the differences between cultivars.

**Figure 7:**
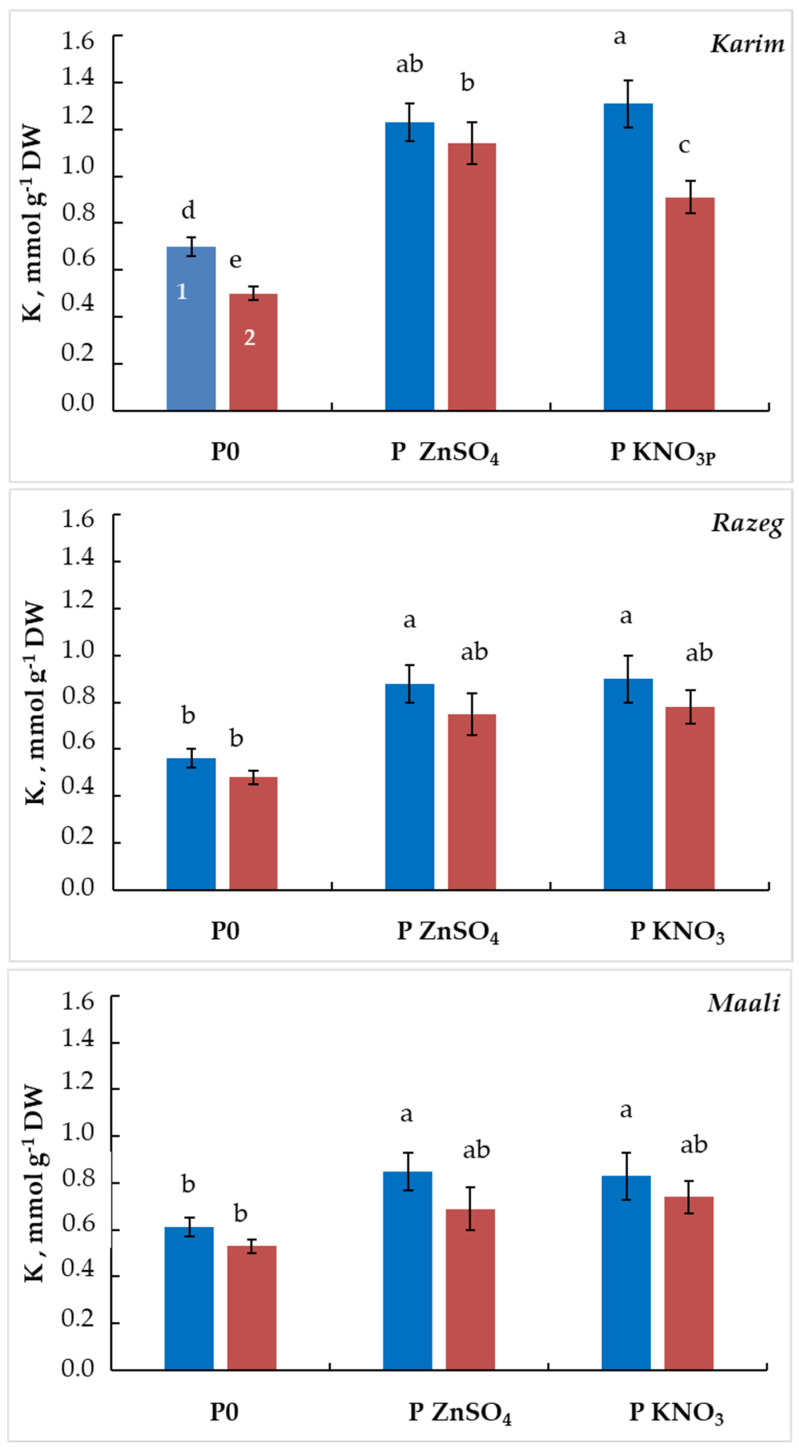
Potassium concentration in non-germinating durum wheat seeds subjected to different salinity stress levels (1: MSS = 5 g L^−1^ NaCl, 2: SSS = 10 g L^−1^ NaCl). The letters in the figure indicate the significance of the differences. Means with different letters are significantly different at α = 0.05 according to Fisher’s Least Significant Difference. The standard error of the mean of five replicates is represented by the bars on the columns. P0: non-primed seeds, P ZnSO_4_: seeds primed with ZnSO_4_, P KNO_3_: seeds primed with KNO_3_.

**Figure 8:**
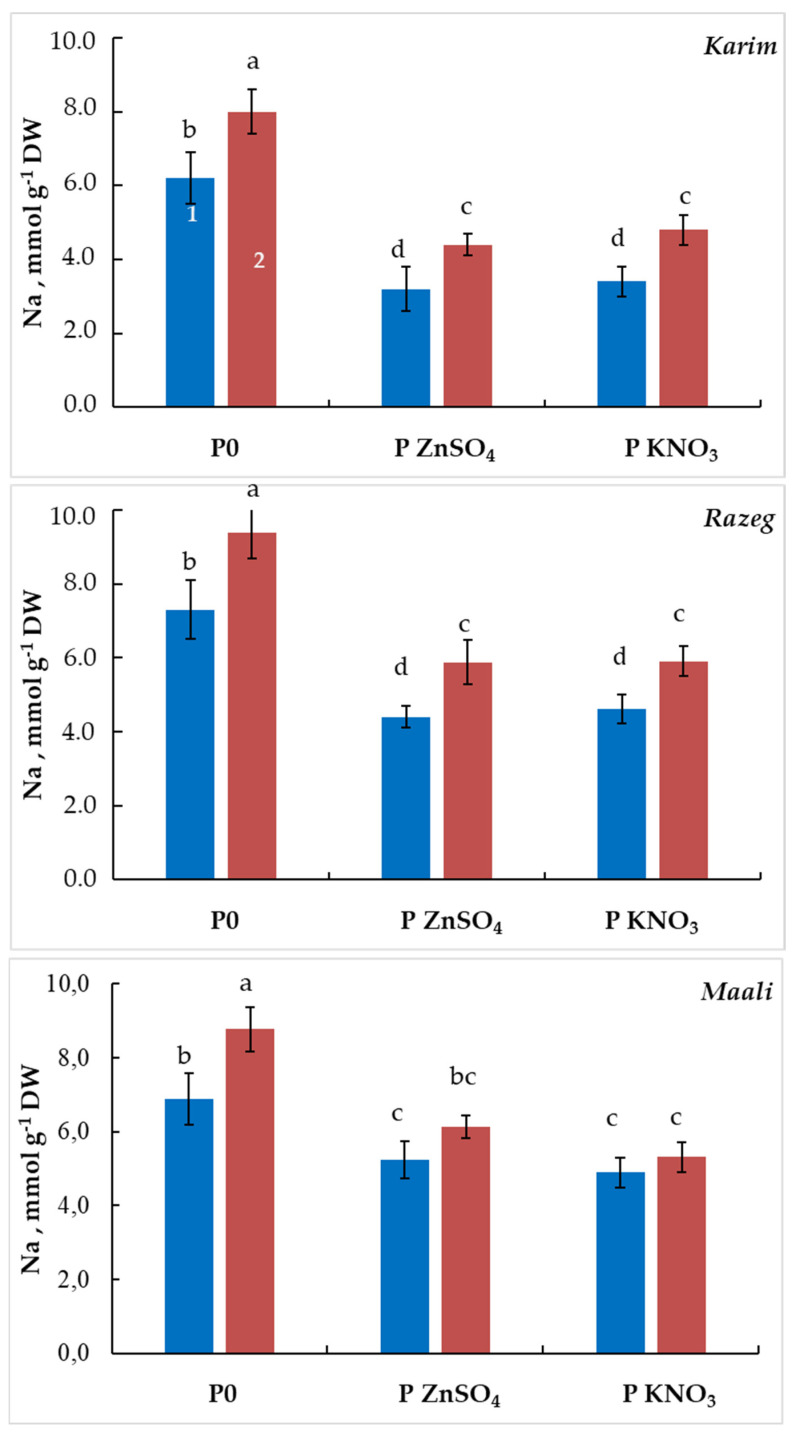
Sodium concentration in non-germinating durum wheat seeds subjected to different salinity stress levels (1: MSS = 5 g L^−1^ NaCl, 2: SSS = 10 g L^−1^ NaCl). The significance of the differences is marked by the letters in the figures. Means with different letters are significantly different at α = 0.05 according to Fisher’s Least Significant Difference. The standard error of the mean of five replicates is represented by the bars on the columns. P0: non-primed seeds, P ZnSO_4_: seeds primed with ZnSO_4_, P KNO_3_: seeds primed with KNO_3_.

Although the beneficial effect of seed priming by ZnSO_4_ or KNO_3_ is certain in this study, the mechanism by which it alleviates the effect of salt stress on germination remains to be determined. In this regard, we analyzed sodium and potassium concentrations in non-germinating seeds. Figure 7 shows that K concentration increased by 76% and 87% in Karim, 57% and 61% in Razeg, and 39% and 36% in Maali under MSS, and by 128% and 82% in Karim, 56% and 63% in Razeg, and 30% and 40% in Maali under SSS when primed with ZnSO_4_ and KNO_3_, respectively. For the cultivar differences, Karim accumulates 25% and 15% more K than Razeg and Maali under MSS, while no difference is observed under SSS. When primed with ZnSO_4_, Karim accumulates 40% and 45% more K than Razeg and Maali, respectively, under MSS and 52% and 65% more K than Razeg and Maali, respectively, under SSS. In contrast, sodium analysis demonstrated that wheat seeds accumulated Na at high level in all cultivars incubated under MSS and SSS (Figure 8). However, seed priming significantly decreased the accumulation of this ion independently of salinity level. In fact, Na concentration decreased by 40–50% in Karim, 35–40% in Razeg, and by 25–40% in Maali in non-germinating seeds primed with ZnSO_4_ or KNO_3_. The previously observed cultivar differences were maintained at this level. In non-primed seeds, Karim contains less Na than Razeg and Maali (−10% and −15%, respectively), whereas it reached −23 to −39% when primed with ZnSO_4_ and −10 to −30% when primed with KNO_3_. These results led us to suggest that seed priming with ZnSO_4_ or KNO_3_ allows for the alleviation of salinity stress through adjustment of the ionic balance in germinating seeds. The calculated K/Na ratio (Table 1) showed a substantial increase in primed seeds with respect to cultivar differences, with Karim maintaining its superiority at this level.

**Figure 9:**
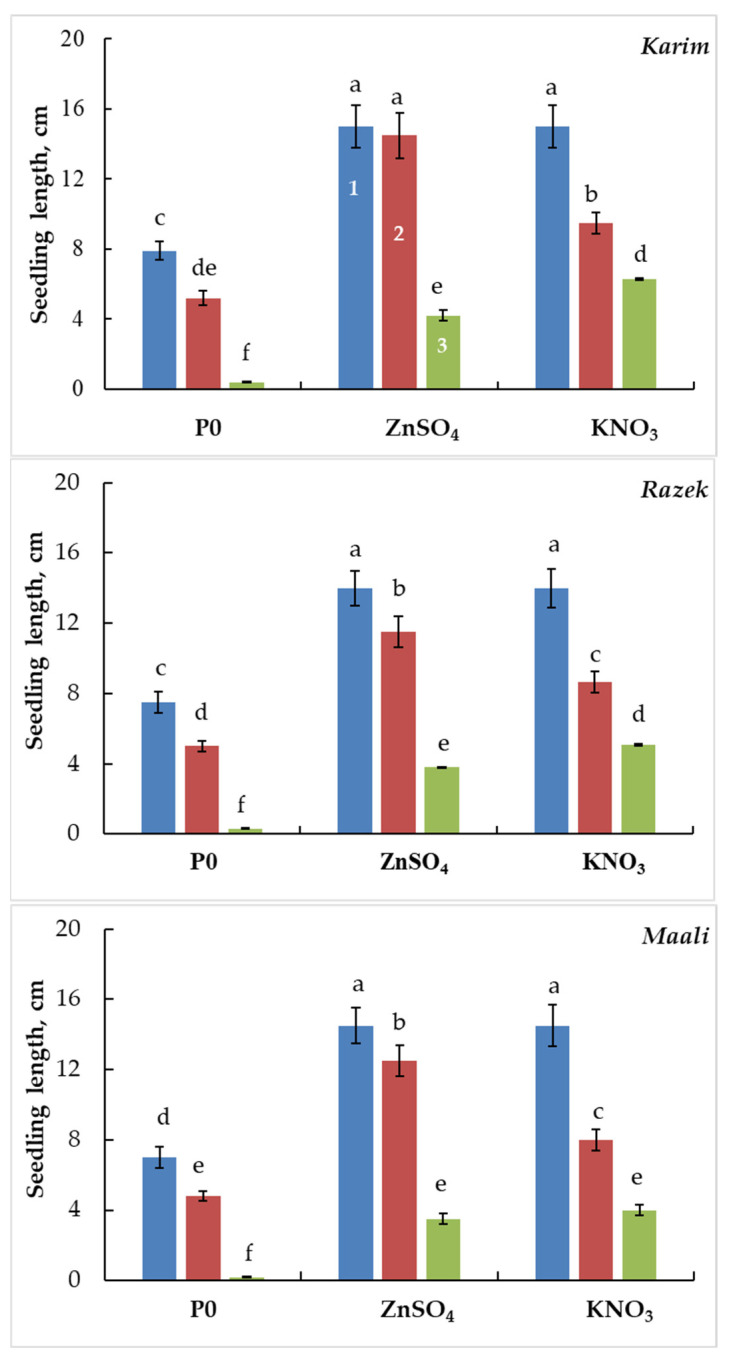
Seedling length of durum wheat subjected to different salinity stress levels for 11 days (4 days for germination and an extra 7 days for emergence). Seeds were primed (or not, P0) with ZnSO_4_ or KNO_3_. The significance of the differences is marked by the letters in the figures. Means with different letters are significantly different at α = 0.05 according to Fisher’s Least Significant Difference. The standard error of the mean of 20 seedlings is represented by the bars on the columns. 1: 0 g L^−1^ NaCl, 2: 5 g L^−1^ NaCl, and 3: 10 g L^−1^ NaCl.

The seedling emergence was recorded 7 days after the germinating phase. Figure 9 shows that salinity stress significantly reduced seedling growth in all cultivars. Under MSS, seedling length decreased by more than 30% when exceeding 90% under SSS. However, seed priming clearly attenuated the deleterious effects of salinity. Following priming with ZnSO_4_, the inhibitory effect on seedling growth declined to 3%, 18%, and 14% under MSS and to 71%, 67%, and 72% under SSS in Karim, Razeg, and Maali, respectively. In seeds primed with KNO_3_, the inhibitory effect on seedling growth declined to 37%, 38%, and 45% under MSS and to 31%, 41%, and 50% under SSS in Karim, Razeg, and Maali, respectively. Thus, we can suggest that seedling establishment is more sensitive than seed germination to salinity stress, and that the most beneficial effect of priming on seedling establishment was gained with ZnSO_4_ under MSS and with KNO_3_ under SSS. Compared to unprimed seeds, seedling length subjected to SSS increased by 11%, 13%, and 18% following seed priming with ZnSO_4_ and by 16%, 17%, and 20% following seed priming with KNO_3_ in Karim, Razeg, and Maali, respectively.

The initial vigor (IV), which relates these two early stages of the plant cycle, demonstrated that IV decreased significantly in all cultivars under salinity stress with respect to the previous differences (Table 1). Under MSS, IV decreased by 40 to 45% in all cultivars when exceeding 95% under SSS. However, seed priming clearly attenuated the deleterious effects of salinity on seedling IV. This parameter decreased following ZnSO_4_ seed treatment by 9%, 25%, and 23% instead of 40 to 45% under MSS, and by 74%, 76%, and 81% instead of more than 95% under SSS in Karim, Razeg, and Maali, respectively. When seeds were primed with KNO_3_, the beneficial effect was observed only under SSS, where IV decreased by 63%, 68%, and 75% instead of more than 95% in Karim, Razeg, and Maali, respectively. Nevertheless, the positive effect of seed priming on seedling establishment clearly appeared when comparing seed-primed seedlings with seed-unprimed seedlings. In control plants, IV increased by two times in seedlings resulting from primed seeds compared to those from unprimed ones in all cultivars (Table 1). Under MSS, IV increased by two times in seedlings resulting from seeds primed with KNO_3_ and by three times in seedlings resulting from seeds primed with ZnSO_4_. Under SSS, the beneficial effect of seed priming was expressed even more in all cultivars, being 13, 17, and 21 times IV higher in seed ZnSO_4_-primed seedlings as compared to unprimed and 18, 23, and 28 times IV higher in seed KNO_3_-primed seedlings compared to unprimed in Karim, Razeg, and Maali, respectively.

Finally, we analyzed the sensitivity tolerance index (STI) based on germination capacity, seedling growth, and initial vigor. As Table 2 shows, independent of salinity stress severity and the parameter used to calculate STI, Karim expressed higher tolerance than either Razeg and Maali. Increasing severity of salinity decreased STI in all cultivars with respect to the above differences. In fact, GC-dependent STI decreased by 16% in Karim, 21% in Razeg, and 22% in Maali when subjected to SSS as compared to MSS (Table 2). When STI depends on seedling growth or IV, it decreases by more than 90% under SSS as compared to MSS in all cultivars. Otherwise, seed priming significantly increases wheat tolerance to salinity stress by increasing germination capacity and seedling growth, with a subsequent positive effect on initial vigor. The beneficial effects of ZnSO_4_ and KNO_3_ seed priming appear under MSS (an STI IV-based increase of 4 to 6 times compared to unprimed seeds) and are particularly apparent under SSS (an STI IV-based increase of 25 to 60 times compared to unprimed seeds).

## 3. Discussion

It is well established that the germination process is among the most susceptible stages to environmental stresses, including salinity [21], and durum wheat is no exception. Even in irrigated systems, wheat is subjected to salt stress because of the quality of the irrigation water (2.7 g L^−1^ in the irrigated perimeter of Sidi Bouzid where the study took place). Through its adverse impacts on the plant’s metabolic and functional structure, salinity stress significantly hampers plant growth, development, and yield [22,23,24]. Such effects during early phases of the plant cycle lead to poor germination, seedling establishment, and seedling vigor, which certainly have impacts on the subsequent phases of the plant cycle. However, seed priming has been documented as a useful technique owing to the resulting ability of crops to grow, endure, and thrive in saline environments [12,25,26,27]. In the present study, we observed reduced GC, DMG, Vc, seedling length, and initial vigor, which is accentuated with the severity of stress. A number of cultivar differences were observed, with Karim showing better tolerance compared to Razeg and Maali. In contrast, MTG increased under salinity stress, particularly under SSS. However, seed halopriming with ZnSO_4_ or KNO_3_ allowed the mitigation of such impacts on germination and seedling establishment traits to a great extent, and was almost able to recover the normal GC even under SSS. In fact, the negative effect of salts can be explained by a slowdown in reserve mobilization due to the inactivation of synthesis of enzymes for starch hydrolysis and/or inhibition of the transfer of hydrolysis products from the endosperm to the embryo [28]. During water or salt stress, late embryogenesis abundant proteins (LEA) and heat shock proteins (HSP) accumulate [29]. The latter act as osmoprotectants and antioxidants [30]. Other studies have indicated that salt stress induces the production of reactive oxygen species (ROS), leading to the peroxidation of chloroplast and mitochondrial lipids, loss of membrane integrity, protein degradation, and enzyme inactivation, while osmopriming induces production of antioxidants [17,19,30,31,32]. Thus, the beneficial effect of seed pretreatment observed in this study can be explained by the ability of the employed agents (KNO_3_ and ZnSO_4_) to mitigate these salinity impacts. Furthermore, these results are comparable with earlier studies on numerous priming agents performed in crops. In fact, halopriming involves the soaking of seeds in aerated solutions of inorganic salts such as KNO_3_ and MgSO_4_, in addition to others (NaCl, KCl, CaCl_2_, K_3_PO_4_, etc.), prior to stress exposure. It is an eco-friendly technique that activates signaling molecules and enhances the inherent salt tolerance potential in plants, which helps in recovery from salt-induced damages [33,34]. In the present study, we demonstrated that seed pretreatment with KNO_3_ and ZnSO_4_ significantly increased K accumulation in seeds against reduced Na uptake (Figure 7 and Figure 8), leading to an increased K/Na ratio (3 to 4 times in Karim, 2.5 times in Razeg, and 2 to 2.3 times in Maali when primed with ZnSO_4_ and KNO_3_, respectively). Thus, it is clear that seed halopriming allows for the alleviation of salinity stress through improved ionic equilibrium and water uptake due to K accumulation as well as through reduced Na toxicity and its diverse effects on the cell metabolism. Accordingly, other authors have reported that halopriming can improve the K^+^/Na^+^ ratio and Ca^2+^ absorption under salinity stress, which helps to retain ion homoeostasis, improve seedling growth, increase photosynthetic activity, and reduce electrolyte leakage in crop plants [35]. In *Vigna radiata,* halopriming of seeds with 50 mM NaCl and subsequent exposure to the same dose has been reported to facilitate ion adjustments and alter organic acid production [36]. Biswas et al. [37] reported similar behavior with regard to ion uptake associated with improved growth in *Cajanus cajan* and *Vigna mungo* seeds pretreated with NaCl and then exposed to 50, 100, and 150 mM salt. In rice, Sen and Puthur [38] demonstrated that pre-soaking seeds with NaCl accelerated the accumulation of certain metabolites (sugars, phenolics, free amino acids, proline, ascorbate, glutathione, etc.) and increased ROS scavenging enzymes that promote salinity tolerance (superoxide dismutase, catalase, ascorbate peroxidase, and glutathione peroxidase). Similar results have been reported on seed halopriming with NaCl to alleviate the inhibitory effect of salinity on nitrogen metabolism and DNA biosynthesis in different plant species [39,40]. Recent reports suggest the ameliorative effect of calcium on plants under salinity may be associated with the maintenance of an optimal Na^+^/K^+^ ratio in the cytoplasm. In *Cucumis sativus* seed, priming with CaCl_2_ conferred salt tolerance by enhancing proline accumulation [41]. The same priming agent promoted *Sorghum bicolor* seed germination under 150 mM NaCl [42].

Our results show that salt stress induced an osmotic stress inhibitory effect on germination that dominated under MSS and a toxic stress that dominated under SSS. Cultivars differed in the extent of the toxic effect. The osmotic stress can be overcome by simple dilution, while toxicity stress is difficult to control without effective intervention. However, seed priming with KNO_3_ or ZnSO_4_ attenuated these two types of stress and prevented their effects, allowing a maximum FGC to be achieved following the priming treatments. Munns et al. [43] reported that severe salinity stress in cotton plants disrupts the osmotic balance and water uptake. Ions excluded by the cell membrane and overaccumulating in the cell walls (Na^+^, Cl^−^, …etc.) can cause cellular dehydration [44]. For these reasons, the metabolic mechanisms that govern osmotic stress caused by salinity are similar to those caused by drought stress [45]. In contrast, the toxic effect is explained by the absorption of sodium and chloride, and possibly other ions, that surpass the physiological limits in some way. The overaccumulated Na^+^, Cl^−^, and even other ions cause cell injury and inhibit germination due to disrupted cell division and elongation. Injury occurs when the accumulated ions exceed the cell’s capacity to compartmentalize them in the vacuole [46]. Consequently, the enzyme activity in the cytoplasm is inhibited [47] and the initiation of the radicles does not take place. Zang et al. [48] reported that excessive accumulation of Na^+^ and Cl^−^ disrupts the cytoplasmic ion balance, particularly that of Ca^2+^. Na^+^ can displace bound Ca^2+^ in the plasma membrane, damaging its structural integrity and functionality. Considering the benefits of the above-discussed halopriming agents and the present results, we can suggest that the beneficial effect of seed priming with KNO_3_ or ZnSO_4_ consists in its ability to adjust the absorption of Na, Cl, and nutrients to levels that are physiologically compatible with ordinary cellular metabolism. The observed accumulation of K^+^ in response to priming (Figure 7) and the cultivar relationship between the extent of K^+^ accumulation and the extent of the toxicity effect (Figure 6; Karim accumulated more K^+^ in response to priming, and correspondingly had a lesser toxicity effect) is consistent with this interpretation. In fact, it is well established that potassium is the most preponderant cation in plant cells, playing a key role in osmoregulation [49,50]. 

The improved seedling establishment and growth observed in the present study as a result of seed priming with KNO_3_ and ZnSO_4_ is not an exception to previous studies in halophytes and glycophytes, though there are some differences. Ben Youssef et al. [51] conducted a comparative study between a halophyte (*Hordeum maritimum*) and a glycophyte (*Hordeum vulgare*) and showed that salt stress affected seedling development of both species, with a lesser effect on the halophyte variety. The final germination rate, seedling length, dry biomass, and mineral content were hampered by salinity stress, with a higher effect on the glycophytic variety. However, the beneficial effect of halopriming was more clearly revealed in the latter variety. The effective role of KCl priming under salinity has been well documented as well. In *Zea mays*, seeds pre-soaked with KCl showed improved germination percentage, germination rate index, germination coefficient, and seedling vigor under 20, 30, 40, and 50 mM NaCl stress compared to unprimed seedlings [52]. *Capsicum annuum* seed priming with KCl is thought to contribute to salt tolerance by promoting proline accumulation under 2, 4, 6, 8, 10, and 12 g l^−1^ NaCl treatments [53]. Farhoudi and Lee [54] reported that priming corn seeds with KNO_3_ increased seedling emergence, seedling dry weight, -amylase activity, and carbohydrate metabolism when decreasing lipid peroxidation under salinity stress. In maize and wheat seeds primed with KNO_3_, the conferred tolerance to salt stress was revealed through improved germination indices, osmolyte accumulation, and antioxidant enzyme activities [20,55]. In wheat, Khan et al. [19] revealed that seed priming with ZnSO_4_ improved plant growth and enzyme-dependent antioxidant activity. Aboutalebian et al. [56] stated that priming with ZnSO_4_ had a strong positive effect on the speed and emergence of wheat seeds grown under rainfed conditions. In addition, Tajlil et al. [57] reported an enhancement of biochemical parameters and seed germination in chickpeas with zinc sulfate priming. Bourhim et al. [58] reported that quinoa seed priming with ZnSO_4_ improved the germination parameters, germination speed, and final germination percentage by more than 100%. In the field, seed priming is important even for the post-germination stages. Many authors have shown that seed pretreatment allows for acceleration and synchronization of germination in different species of field crops, including beans, lentils, wheat, maize, rice, watermelon, melon, tomato, carrot, and amaranth [59], as well as better growth, earlier flowering, greater stress tolerance, and higher yield [60,61,62].

Taken together, our results demonstrate that salinity stress significantly inhibited germination and seedling traits in all studied wheat cultivars, though with a number of differences. The Karim cultivar showed better tolerance to this abiotic stress. Under MSS, OE dominates in Karim, while TE dominates in Razeg and Maali. Under SSS, the TE effect dominates in all cultivars, with this dominance being lesser in Karim. However, the most important result that emerges from this study is the beneficial effect of halopriming in alleviating the harmful effects of salinity toxicity stress and the cultivar differences in response to salt toxicity and seed priming. KNO_3_ and ZnSO_4_ effectively alleviated the inhibitory effect of salt stress on seed germination and seedling establishment while significantly improving the initial vigor, which would certainly have a positive impact on the subsequent stages of plant development. The improved K/Na ratio observed in this study suggests that halopriming improves potassium uptake at the expense of sodium, exerting a positive impact on water absorption and the protection of membranes and cell structures. As compared to Maali and Razeg, the Karim durum wheat cultivar showed a higher ability to respond to salt stress and seed priming. This cultivar showed greater tolerance to salinity outside of any pre-treatment, and responded better to priming. The calculated STI clearly discriminated the studied cultivars and further confirmed the tolerance of Karim. The total recovery of germination capacity and seedling emergence, even under SSS, suggests very powerful mechanisms that relieve the harmful effects of salinity. Ionic compartmentation, osmolite accumulation, and enzymatic and non-enzymatic antioxidant capacity are good candidates for these mechanisms, and are programmed for our next studies.

## 4. Materials and Methods

### 4.1. Biological Material and Experimental Design

Three Tunisian durum wheat cultivars provided for us by the High Institute of Field Crops of Kef (Karim, Maali, and Razeg) were used in this study. Salinity treatment was applied as moderate (MSS) and severe (SSS) salinity stress during germination and early-stage plant establishment. Seeds were prescreened for their health and homogeneity and were used at the rate of hundred seeds for each cultivar and treatment (twenty seeds per Petri dish containing filter paper; five dishes per treatment and cultivar). For control plants, seeds were germinated in deionized water (control—0 salt), whereas stressed ones were germinated in NaCl solution at a rate of 5 g L^−1^ (MSS) or NaCl solution at a rate of 10 g L^−1^ (SSS). Seeds were germinated in an incubator with the temperature fixed at 27 °C.

Germination percentage (GP) was recorded daily; the experiment ended after three successively stable GPs, which were retained as the germination capacity (GC). The presented results are the mean of 100 seeds. To discriminate between the toxic and osmotic parts of the salinity stress effects, we calculated seed germination recovery. To do this, the non-germinating seeds were transferred to deionized water for an additional three days and the germination percentage was recorded as previously. For the halopriming tests, we soaked the seeds in solutions containing KNO_3_ (500 mg L^−1^) or ZnSO_4_ (500 mg L^−1^) for 6 h, then transferred them to new Petri dishes containing the above NaCl concentrations (5 and 10 g L^−1^).

The germinated seeds from each treatment were maintained for an extra week to highlight the seedling growth. They were transferred to a greenhouse (Faculty of Sciences and Techniques of Sidi Bouzid, 35°2′7.58″ N, 9°29′2.18″ E) under natural light and a temperature of 25 °C/17 °C (±2 °C, day/night; relative humidity about 75%). This parameter was presented as the length of the seedling (main shoot + root) after 11 days (4 days for germination + 7 extra days).

### 4.2. Experimental Design and Germination, Initial Vigor Indicators

The experimental design consisted of two factorials (genotype × NaCl concentration) arranged in a completely randomized design with 5 replications of 20 seeds in the germination stage (results presented as means of 100 seeds) and 20 replications in the seedling stage (main shoot and root size measured in 20 seedlings). The following traits were determined based on the various experiments:

- Germination capacity (GC, %): This parameter represents the maximum germination percentage reached at the end of the experiment (three stable GPs), expressed as follows:
  $$GC=\frac{b}{a}\times 100$$
  where a = total number of used seeds and b = maximum number of germinated seeds.
- Germination percentage (GP, %): Expressed as the ratio of germinated seeds at day *n* to the total number of seeds:
  $$GP=\frac{c}{a}\times 100$$
  where a = total number of seeds, c = number of cumulative germinated seeds at day *n*, *n* = day 1, 2, … *n*.
- Velocity coefficient (Vc): Calculated as follows:
  $$Vc=\frac{\left(N1+N2+N3\ldots +Nn\right)\times 100}{\left(N1T1+N2T+N3T\ldots +NnTn\right)}$$
  where Nn represents the number of germinated seeds between time Tn−1 and Tn.
- Mean time germination time (MTG, days): This parameter is determined according to the following formula [47]:
  $$MTG=\left[\frac{\sum ni\times di}{b}\right]$$
  where n represents the number of seeds germinated on day i, d represent the incubation period in days, and b is the total number of seeds germinated upon treatment.
- Daily mean germination (DMG, %): According to Osborne and Mercer [63], the DMG is calculated as the ratio of the germination percentage to the total number of germination days at the end of the experiment:
  $$DMG=\frac{b}{n}\times 100$$
  where b = maximum number of germinated seeds upon treatment and n = number of germination days at the end of the experiment.
- Germination recovery (GRec, %): Expressed as the capacity to recover the germination capacity lost on NaCl treatment, calculated using the following equation:
  $$GRec=\frac{d}{\left(a-b\right)}\times 100$$
  where d is the number of seeds germinated after transfer in deionized water, b is the maximum number of seeds germinated under salinity, and a represents the total number of seeds [64].
- Final germination capacity (FGC, %): This parameter represents the maximum germination capacity reached after recovery, expressed as the sum of GC and GRec at each treatment:
  $$FGC=\frac{\left(b+d\right)}{a}\times 100$$
  where a, b, and d are defined as above.
- Initial vigor (IV): This indicator is a good parameter for relating the germination capacity to plant growth. It is calculated as follows:
  IV=GC×SL
  where GC = germination capacity and SL = seedling length.
- Stress index (SI): This parameter is the indicator of the degree of stress on each cultivar. It is calculated as follows:
  (a) based on GC:
    $$SI-GC=1-\frac{GCs-GCc}{GCc}$$
  (b) based on plant growth:
    $$SI-DW=1-\frac{DWs-DWc}{DWc}$$
    where GCs represents the germination capacity of seeds subjected to salt stress, GCc represents the germination capacity of control seeds, DWs means the dry weight produced by salt-stressed plants, and DWc means the dry weight produced by control plants.

### 4.3. Analysis of Sodium and Potassium

For chemical analysis, five batches from non-germinating seeds of each treatment (MSS and SSS) and cultivar were dried at 70 °C for 72 h and ground to fine powder. Ion extraction was carried out in 0.5% HNO_3_, and K and Na were measured using a flame photometer (JENWAY, model PFP7, Suffolk Dr Chelmsford, UK).

### 4.4. Statistical Analysis

The experiment was conducted factorially with treatments arranged in a completely randomized design. Results are presented as mean ± standard error. To determine whether the different experimental factors affected germination and/or seedling emergence, analysis of variance (ANOVA) was performed. The significance of differences among treatments was determined by Fisher’s least significant difference test (LSD) (*p* < 0.05). Treatment means were declared significant when the difference between any two treatments was greater than the LSD value generated from the ANOVA. They are marked by different letters in the figures and tables.

## 5. Conclusions

The present work focused on the importance of halopriming in alleviating the adverse effects of salinity on germination and the initiation of vegetative development in durum wheat. Our results show that pretreatment of wheat seeds with ZnSO_4_ or KNO_3_ completely eliminates ET in all cultivars and allows total recovery of germination capacity, even under SSS. These priming agents improve both initial vigor and seedling establishment. ZnSO_4_ and KNO_3_ are useful agents that promote salt tolerance in durum wheat, confirming the effectiveness of halopriming as a useful, effective, rapid, and eco-friendly strategy for saline soil and water valorization.

## Figures and Tables

**Table 1:** Effect of salinity stress on the K/Na ratio in non-germinating seeds and the Initial Vigor (IV) of durum wheat cultivars subjected to different salinity stress levels. Fisher’s least significant difference (*n* = 5 for K/Na ratio, and *n* = 100: 5 replicates of 20 seeds for IV) was used to test the significance of the means ± standard errors differences. Within rows, the same letter indicates that the means are not significantly different at α = 0.05. K/Na-ZnSO_4_: seeds primed with KNO_3_, K/Na-ZnSO_4_: seeds primed with ZnSO_4_, IV: seeds non-primed, IV-ZnSO_4_: seeds primed with ZnSO_4_, IV-KNO_3_: seeds primed with KNO_3_.

Cultivar	NaCl, g L^−1^	K/Na-ZnSO_4_	K/Na-KNO_3_	IV	IV-ZnSO_4_	IV-KNO_3_
Karim	0			743 ± 52 ^e^	1440 ± 71 ^a^	1410 ± 88 ^a^
	5	3.4 ± 0.21 ^b^	3.4 ± 0.28 ^b^	447 ± 33.7 ^h^	1305 ± 63 ^b^	836 ± 55 ^d^
	10	4.1 ± 0.32 ^a^	3.0 ± 0.25 ^c^	29 ± 1.68 ^l^	370 ± 27 ^i^	517 ± 34 ^g^
Razeg	0			690 ± 45.6 ^ef^	1288 ± 72 ^b^	1316 ± 78 ^b^
	5	2.6 ± 0.24 ^d^	2.5 ± 0.20 ^d^	380 ± 22.8 ^i^	966 ± 66 ^c^	796 ± 42 ^d^
	10	2.5 ± 0.19 ^d^	2.6 ± 0.22 ^d^	18 ± 1.23 ^m^	312 ± 21 ^j^	418 ± 22 ^hi^
Maali	0			602 ± 46.7 ^f^	1305 ± 83 ^b^	1305 ± 77 ^b^
	5	1.8 ± 0.11 ^e^	1.9 ± 0.14 ^e^	355 ± 25.7 ^ij^	1000 ± 63 ^c^	640 ± 39 ^f^
	10	1.9 ± 0.16 ^e^	2.3 ± 0.18 ^de^	12 ± 1.0 ^n^	245 ± 27 ^k^	320 ± 28 ^j^

**Table 2:** Stress tolerance index (STI) calculated based on germination capacity, seedling length, and initial vigor in durum wheat cultivars subjected to salinity stress. Fisher’s least significant difference (*n* = 100, 5 replicates of 20 seeds) was used to test the significance of the means ± standard errors differences. Within rows, the same letter indicates that the means are not significantly different at α = 0.05. P_0_: seeds non-primed, P_ZnSO_4__: seeds primed with ZnSO_4_, P_KNO_3__: seeds primed with KNO_3_.

		STI-Germination Capacity			STI-Seedling Growth			STI-Initial Vigor		
	NaCl, g L^−1^	P_0_	P_ZnSO_4__	P_KNO_3__	P_0_	P_ZnSO_4__	P_KNO_3__	P_0_	P_ZnSO_4__	P_KNO_3__
Karim	5	145 ± 10.2 ^a^	155 ± 10 ^a^	148 ± 7.5 ^a^	0.74 ± 0.05 ^j^	3.90 ± 0.25 ^a^	2.55 ± 0.17 ^d^	5951 ± 135 ^l^	33,677 ± 324 ^a^	21,125 ± 288 ^d^
	10	121 ± 9.6 ^b^	151 ± 9.4 ^a^	138 ± 9.1 ^ab^	0.06 ± 0.003 ^m^	1.13 ± 0.09 ^h^	1.69 ± 0.11 ^f^	383 ± 26.3 ^p^	9538 ± 189 ^i^	13,054 ± 235 ^g^
Razeg	5	125 ± 8.4 ^b^	138 ± 8.6 ^ab^	155 ± 8.6 ^a^	0.67 ± 0.05 ^k^	2.9 ± 0.18 ^c^	2.17 ± 0.14 ^e^	4699 ± 202 ^n^	22,298 ± 313 ^c^	18,768 ± 168 ^e^
	10	99 ± 6.3 ^d^	135 ± 8.8 ^ab^	138 ± 7.7 ^ab^	0.04 ± 0.002 ^m^	0.95 ± 0.07 ^i^	1.28 ± 0.11 ^g^	223 ± 17.6 ^q^	7192 ± 211 ^k^	9863 ± 189 ^h^
Maali	5	114 ± 7.7 ^c^	129 ± 9.2 ^b^	129 ± 7.2 ^b^	0.60 ± 0.004 ^l^	3.25 ± 0.21 ^b^	2.08 ± 0.13 ^e^	3832 ± 186 ^o^	23,387 ± 334 ^b^	14,968 ± 221 ^f^
	10	89 ± 5.8 ^e^	113 ± 7.8 ^c^	129 ± 6.5 ^b^	0.03 ± 0.001 ^m^	0.91 ± 0.06 ^i^	1.04 ± 0.09 ^hi^	125 ± 9.2 ^r^	5730 ± 157 ^m^	7484 ± 193 ^j^

## Data Availability

The data is contained within the manuscript.
